# Genome-wide linkage using the Social Responsiveness Scale in Utah autism pedigrees

**DOI:** 10.1186/2040-2392-1-8

**Published:** 2010-04-08

**Authors:** Hilary Coon, Michele E Villalobos, Reid J Robison, Nicola J Camp, Dale S Cannon, Kristina Allen-Brady, Judith S Miller, William M McMahon

**Affiliations:** 1 Utah Autism Research Project, Department of Psychiatry and Division of Genetic Epidemiology, University of Utah, 650 Komas Drive, Suite 206, Salt Lake City, UT 84108, USA

## Abstract

**Background:**

Autism Spectrum Disorder**s **(ASD) are phenotypically heterogeneous, characterized by impairments in the development of communication and social behaviour and the presence of repetitive behaviour and restricted interests. Dissecting the genetic complexity of ASD may require phenotypic data reflecting more detail than is offered by a categorical clinical diagnosis. Such data are available from the Social Responsiveness Scale (SRS) which is a continuous, quantitative measure of social ability giving scores that range from significant impairment to above average ability.

**Methods:**

We present genome-wide results for 64 multiplex and extended families ranging from two to nine generations. SRS scores were available from 518 genotyped pedigree subjects, including affected and unaffected relatives. Genotypes from the Illumina 6 k single nucleotide polymorphism panel were provided by the Center for Inherited Disease Research. Quantitative and qualitative analyses were done using MCLINK, a software package that uses Markov chain Monte Carlo (MCMC) methods to perform multilocus linkage analysis on large extended pedigrees.

**Results:**

When analysed as a qualitative trait, linkage occurred in the same locations as in our previous affected-only genome scan of these families, with findings on chromosomes 7q31.1-q32.3 [heterogeneity logarithm of the odds (HLOD) = 2.91], 15q13.3 (HLOD = 3.64), and 13q12.3 (HLOD = 2.23). Additional positive qualitative results were seen on chromosomes 6 and 10 in regions that may be of interest for other neuropsychiatric disorders. When analysed as a quantitative trait, results replicated a peak found in an independent sample using quantitative SRS scores on chromosome 11p15.1-p15.4 (HLOD = 2.77). Additional positive quantitative results were seen on chromosomes 7, 9, and 19.

**Conclusions:**

The SRS linkage peaks reported here substantially overlap with peaks found in our previous affected-only genome scan of clinical diagnosis. In addition, we replicated a previous SRS peak in an independent sample. These results suggest the SRS is a robust and useful phenotype measure for genetic linkage studies of ASD. Finally, analyses of SRS scores revealed linkage peaks overlapping with evidence from other studies of neuropsychiatric diseases. The information available from the SRS itself may, therefore, reveal locations for autism susceptibility genes that would not otherwise be detected.

## Background

Compelling evidence exists to suggest that autism spectrum disorder (ASD) has a complex heterogeneous genetic aetiology [1]. However, the specific nature of the genetic aetiology is not well understood. This genetic heterogeneity is, perhaps, not surprising given the phenotypic complexity and range of expression of ASD. Examining autism-related characteristics along a continuum of expression may yield more insight into the particular roles played by autism susceptibility genes. In addition, if ASD characteristics are viewed on a continuum, then clinically unaffected family members can also contribute information to family genetic studies.

Relatives of individuals with ASD have subclinical traits related to autism more frequently than the relatives of controls [2-8]. These subclinical traits can vary in intensity and have been collectively termed the broader autism phenotype (BAP). In addition, characteristics of clinically affected subjects - such as language delay, language deficits, social deficits, repetitive behaviours and insistence on sameness - have been successfully used to refine and clarify genetic studies of autism [9-12]. While categorical diagnostic status has been used successfully to identify autism linkage regions [1], quantitative measures that include the BAP provide additional information from clinically unaffected family members, and also provide more detailed phenotypic information on affected subjects.

The social, behavioural and communication characteristics of ASD are common and may be viewed as on a continuum with possible cutoffs for affected individuals [13,14]. The social responsiveness scale (SRS) is a quantitative measure of autistic traits with comparable child and adult versions, ranging continuously from significantly impaired to above average [13,15,16]. Several studies have reported significant familial correlations of subclinical features of autism as measured by the SRS [17-19], consistent with previous evidence of significant heritability of the BAP [7]. One previous genome-wide linkage scan has been done using SRS scores obtained from children in 99 nuclear families from the Autism Genetic Resource Exchange [20]. That study included 408 microsatellite markers and two suggestive genetic loci were identified on chromosomes 11 and 17, with other possible regions identified on chromosomes 4, 8, and 10.

We report genome-wide SRS linkage results using single nucleotide polymorphism (SNP) data genotyped on a unique resource of large extended and smaller multiplex ASD families from Utah. We have previously reported linkage evidence in these families using clinical diagnostic status and only including information from affected pedigree members. The current study has two advantages. First, we include additional phenotype information above and beyond our previous affected-only genome scan by using the quantitative SRS measure: clinically unaffected relatives can contribute information to this analysis. The SRS phenotype therefore adds to our previous study in our extended pedigrees. Second, we add to other previous studies of the SRS which focus on nuclear families. The likely heterogeneity of ASD increases the value of studies that include extended pedigrees [21,22]. Collections of small families may include cases with many different susceptibility loci. Subjects affected with ASD who are members of a large extended family may be more likely to share the same genetic causes through their common ancestors. Particularly for rare disease-causing variation, the type of variation most likely underlying autism spectrum disorders, extended pedigrees have substantially more power to reveal true findings that designs with single cases or small families [23]. Once variation has been found, pedigree members can reveal penetrance of the variant and specific associations with related phenotypes. The study of the SRS in pedigrees may be of particular interest, as recent evidence suggests differences in the SRS between simplex and multiplex families [24].

## Methods

### Subjects

Subjects were initially ascertained from 70 pedigrees that had at least two family members with an ASD. Six families did not have sufficient SRS phenotype information (defined as at least two family members with SRS scores) and were therefore dropped from this analysis. A total of 629 genotyped subjects from 64 families were used here, 181 of whom were defined as having either a strictly defined autistic disorder (AD) or a more broadly defined ASD. Table 1 gives the characteristics of these families which include 19 large extended pedigrees (6-9 generations), six families of moderate size (4-5 generations) and 39 smaller families (2-3 generations). The 19 extended pedigrees were identified or confirmed using the Utah Population Database (UPDB), a computerized genealogy database that contains family history information for over 6.5 million individuals who are, for the most part, descendants of the nineteenth century Utah pioneers http://www.hci.utah.edu/groups/ppr/. Previous studies have shown low rates of inbreeding within the UPDB [25,26]. Using the UPDB, we were able to identify many distant family relationships between the individuals with ASD that were not known to the subjects or their families. These relationships were kept confidential. This study has ongoing approval from the University of Utah Institutional Review Board (IRB). All adults participating in the research signed IRB-approved informed consent documents. All subjects under the age of 18 signed approved assent documents and their parents or guardians signed approved parental permission documents. Subjects in this study are independent from the Utah subjects who were contributed to the Autism Genome Project and studied by Liu *et al*. [27]. In addition, none of the families in the current analysis were included in the data set studied by Duvall *et al*. [20].

**Table 1:** Description of the Utah autism spectrum disorder (ASD) families.

Type of family	No. of families	Average No. of generations; standard deviation (SD; range)	Total subjects	Average No. of subjects per family; SD (range)	Total ASD subjects	Average No. of ASD subjects per family; SD (range)	Total No. of subjects with SRS	Average No. of subjects per family with SRS (range)
Large (6-9 generations)	19	7.89; 0.66 (6 to 9)	328	17.26; 12.92 (5 to 50)	81	5.22; 2.54 (2 to 9)	254	13.37; 10.32 (4 to 40)

Moderate (4 generations)	6	4; 0.00 (4)	85	14.17; 11.34 (6 to 32)	21	4.00; 3.39 (2 to 9)	70	11.67; 10.21 (3 to 27)

Small (2-3 generations)	39	2.28; 0.46 (2 to 3)	216	5.54; 2.40 (2 to 11)	79	2.05; 0.57 (1 to 3)	194	4.97; 2.11 (2 to 11)

**Full sample**	**64**		**629**		**181**		**518**	

SRS, Social Responsiveness Scale.

### Phenotyping

Families interested in participating were asked to give questionnaire consent, to give initial information regarding possible exclusion criteria and to complete the Social Communication Questionnaire (SCQ) [28]. The SCQ was developed as a parent report measure based on the Autism Diagnostic Interview-Revised [29]. A cutoff score of 15 on the SCQ has been shown to have good sensitivity (0.85) and specificity (0.75) for discriminating ASD from other diagnoses [30]. Subjects were contacted for possible inclusion if their SCQ score was above 15 or if they had a previous diagnosis of ASD. Subjects were excluded if they reported medical conditions known to be associated with autism (tuberous sclerosis, fragile X, neurofibromatosis, congenital rubella or phenylketonia) or evidence of brain injury. To the best of our knowledge, given currently available data, subjects did not have chromosomal anomalies that could confound our findings. If subjects were eligible for the study, they were asked to sign informed consent for DNA and additional assessments. When possible, all subjects with a suspected ASD were then given both the ADI-R and the Autism Diagnostic Observation Schedule-Generic [31] and study diagnoses were made using these assessments. For cases where assessments could not be obtained, the diagnoses were made according to Diagnostic and Statistical Manual of Mental Disorders (DSM)-IV criteria by a psychologist trained in autism assessment (JSM) using all available information (clinical records, other behavioural data, other questionnaire and interview information).

All available subjects were given the SRS. Completed questionnaires were obtained from a total of 523 family members who were genotyped. Completed questionnaires were obtained for 92.1% of index cases, 83.9% of siblings (affected and unaffected), 87.2% of fathers, 78.8% of mothers and 73.7% of more extended relatives (grandparents, great-grandparents). The child version of the SRS was completed for 205 subjects and the adult version was completed by 318 subjects. The child version was filled out by a parent for almost all cases (203/205; 99.02%). The adult version was usually filled out by a spouse (236/318; 74.21%) but parents were raters for adult siblings over age 18 (59/318; 18.6%). Other informants filled out the remainder of the adult SRS forms (23/318; 7.2%). No children were rated by a parent with ASD. However, after surveys were returned, five adult raters were found to have ASD. Scores from these five raters (fathers) on their spouses (mothers) were not used for subsequent analyses, leaving 518 subjects with scores. There were an additional four children whose parent was coded as a poor rater on the ADI for under-reporting of behaviours. While no data regarding rater quality were available for the SRS directly, the similarity of content across the ADI and SRS made ratings done by these parents was suspect. Scores for these subjects were therefore not used in the quantitative analyses of data. For qualitative SRS analysis, data on these subjects were not deleted because possible under-reporting on the SRS did not impact upon the SRS category.

### Genotyping

Genotyping services were provided by the Center for Inherited Disease Research (CIDR) using the 6 K single nucleotide polymorphism (SNP) Illumina Linkage Panel 12, which includes 6090 SNP markers, with an average genetic coverage of 0.65 cM. There were 46 SNPs who were dropped due to poor performance, leaving 6044 SNPs. Data from 653 pedigree subjects passed stringent quality control criteria for initial inclusion in the study [32]. Of the 629 genotyped subjects in 64 families used in this study, 518 of had SRS data.

Eliminating linkage disequilibrium (LD) between markers in linkage studies has been strongly recommended, as false positive results can occur in the presence of LD [33-36]. As these studies have shown, increases in type I error are most dramatic when LD is high and when there are missing genotypes for parents and other connecting relatives. Recommended thresholds of acceptable LD vary, but a pair-wise *r*^2 ^value of 0.05 between SNPs has now been supported with extensive simulation studies [34]. Therefore, prior to linkage analysis, we screened all SNPs for LD using the PLINK software package [37], which recursively removes SNPs in LD within a sliding window. We set a window size of 50 SNPs, shifted the window by five SNPs at each step, and used a variance inflation factor of 1.5, which is equivalent to a multiple correlation coefficient *R*^2 ^of 0.33 regressed simultaneously over all SNPs in the selected window. This *R*^2 ^considers not only the correlations between SNPs but also between linear combinations of SNPs [37] and corresponds in our data to a pair-wise *r*^2 ^of approximately 0.05. This screening for LD removed 1207 SNPs. Also, as part of the validation procedure, we removed 115 SNPs with a minor allele frequency less than 0.10 and 4 SNPs out of Hardy-Weinberg equilibrium. The total number of SNPs left after this phase was 4718.

### Analyses

We used the genetic map provided by CIDR based on the deCODE genetic map [38]. Base pair positions were obtained from the March 2006 human reference sequence (hg18) assembly. The SRS was first treated as a qualitative trait. Published cutoff scores [15] were used to delineate three groups: SRS unaffected (raw score below 54), SRS spectrum (raw score between 54 and 87) and SRS affected (raw score 87 or above). Subjects who were missing SRS scores were coded as unknown.

This qualitative phenotype was tested using MCLINK, a Markov chain Monte Carlo (MCMC) method that allows for multilocus linkage analysis on large extended pedigrees [39]. Using blocked Gibbs sampling, MCLINK generates inheritance matrices for haplotypes of the markers being analysed and estimates the log-likelihood function linkage statistics. This method results in the reconstruction of haplotype configuration for the entire pedigree structure, allowing for analysis of the full genetic information available in the pedigree. MCMC methods therefore maximize the utility of extended pedigree data, as genetic information increases with the number of meioses in a family [40]. Further, the multipoint analysis maximizes the genetic information available from the entire map of SNP genotypes. In multipoint analyses, genetic information increases with the density of the genotypes [41-43] but is not decreased, with exclusion of markers in high linkage disequilibrium [42]. Internally, MCLINK runs the analysis five times to ensure a consistent solution. Results from MCLINK have shown a high degree of similarity to other MCMC linkage methods [44] and also to exact linkage methods and variance components linkage methods as applied to extended pedigrees [45]. MCLINK has been previously used to identify candidate genomic regions for a number of complex diseases in extended pedigrees [44,46-48]. Allele frequencies for the MCLINK analysis were estimated using all of the observed data.

We performed a non-parametric analysis and a general parametric model-based analysis using simple dominant and recessive model parameters that reproduced the reported population frequency of ASD [49]. For the recessive model, the disease allele frequency was set at 0.05; a penetrance of 0.01, 0.01, 0.5 was assigned to those with SRS scores in the spectrum category and 0.0014, 0.0014, 0.8 was assigned to those with SRS scores in the SRS affected category. For the dominant model, the disease allele frequency was set at 0.0025; penetrances were 0.01, 0.5, and 0.5 for those with SRS scores in the spectrum category and 0.0014, 0.8, 0.8 for those with SRS scores in the SRS affected category. Nonparametric methods provide an analytic approach independent of model assumptions. However, for the analysis of extended pedigrees, the parametric approach may be preferable. Parametric analysis provides assumptions about the genotype-phenotype relationship, simplifying the parameter space and allowing for more powerful and efficient analyses without increasing the false positive rate [22,50,51]. The multipoint heterogeneity LOD score (HLOD) allows for a proportion of unlinked pedigrees (1-alpha) and variation in the recombination fraction. The HLOD provided by MCLINK is robust to model misspecification and may reflect the true position of linkage peaks more accurately under conditions of appreciable heterogeneity [47,50,52]. HLOD scores have been shown to be more powerful than homogeneity LOD scores or model-free methods under these conditions [52,53]. The HLOD has been shown to produce scores consistent with other published methods [47,54]. *P*-values from the non-parametric qualitative analyses were converted to LOD scores for ease of interpretation and comparison with the parametric results. We present results in the commonly used framework of significance thresholds proposed by Lander and Kruglyak [55]: LOD score ≥ 1.88 (*P *= 0.0017) represents suggestive evidence and LOD score ≥ 3.30 (*P *= 0.000048) represents significant evidence. We recognize that our study design does not match perfectly with the design that generated these thresholds but they do provide reasonable benchmarks which enable us to organize and present our data. All *P*-values are theoretical, not empirical.

In order to analyse the SRS as a quantitative trait, we first applied a square root transform the SRS data to adjust for non-normality in the distribution, as has been done with previous SRS analyses [17]. The effects of age and gender were partialled out using regression. MCLINK was used to perform parametric quantitative trait analysis. In a quantitative trait analysis there is no concept of 'affected' or 'unaffected'; all individuals with a trait value are included in the analysis, other individuals are considered unknown. The MCLINK nonparametric analysis considers quantitative similarity among relatives and has been previously described [56]. For the parametric quantitative analysis, the penetrance vector for an individual is based on their observed trait value and three specified trait distributions for the three genotypes at the disease locus (no risk allele, one risk allele, two risk alleles). The three trait distributions are unknown, but can be estimated from the observed trait data. The SRS score cutoffs provide theoretical categories of unaffected, spectrum and affected subjects. Here we considered these three categories as a representing a hypothetical genetic model of: no risk allele, one risk allele and two risk alleles, and means and standard deviations of adjusted SRS scores were estimated. The three distributions for zero, one or two risk alleles were specified to be normal distributions with means of -1.552, 1.184, and 2.558 and standard deviations of 2.238, 1.581, and 1.356, respectively. The penetrance vector for an individual is simply the relative probabilities of the trait being observed from the three distributions. Only the relative probability between the three penetrance values is important as the linkage statistic is a likelihood ratio. For efficient calculation of parametric quantitative LOD scores, the continuous penetrance function is discretized into multiple classes (here, 40) and all individuals with trait values residing in a class are assigned penetrance vectors at the mid-point of the class. Classes were defined starting -3SD below the mean of the zero risk alleles distribution and adding equal increments to reach +3SD above the mean of the two risk alleles distribution.

In summary, we note that use of the SRS results in three important differences between this analysis and our previous affected-only analysis using clinical diagnosis on these families. First, this analysis includes information from an additional 337 clinically unaffected relatives. Second, for the qualitative analysis, affection status was defined using SRS data rather than clinical measures. Third, a quantitative analysis uses a full range of scores on autism-related traits in all measured pedigree members.

## Results

Table 2 describes the diagnostic information for affected subjects. Of the 181 total affected subjects included in this analysis, 165 had data on both the ADI-R and ADOS-G; 16 other cases were assigned a diagnosis based on criteria specified in *Diagnostic and Statistical Manual of Mental Disorders (*DSM-IV-TR criteria). One hundred and thirty-five affected subjects met criteria for strictly defined autistic disorder (AD) and 46 met criteria for an ASD. There was a 7.4:1 male:female ratio among the subjects with strictly defined autism, which fell to 3.6:1 among the subjects with an ASD. For all subjects combined, the male:female ratio was 5.96:1. As expected, ADI-R scores were significantly higher for the autism group compared to the ASD group (*t *= 8.19, *P *< 0.0001 for social; *t *= 6.95, *P *< 0.0001 for verbal; *t *= 2.95, *P *= 0.004 for restricted interests/repetitive behaviours). The nonverbal total cannot be compared because no ASD subjects were nonverbal. Quantitative scores on the ADOS are not compared because of differences between modules but significantly more individuals in the autism group were administered Modules 1 and 2 compared to the ASD group.

**Table 2:** Descriptive information for pedigree subjects.

						Mean ADI domain scores (SD; *N*)			
**Diagnostic group**	***N***	**Male: female**	**IQ > 70 (%)**	**Mean age (SD)**	**Mean SRS score (SD; *N; *% child version)**	**Social**	**Verbal**	**Non-verbal**	**Restr/** **Repet**

Autism*	135	119:16 (7.44:1)	69/125 (55.2)	13.87 (11.44)	107.0 (31.4; *N *= 126; 75.4%)	21.8 (6.4)	17.2 (4.7)	12.3 (2.3; *N *= 26)	6.7 (2.6)

ASD†	46	36:10 (3.60:1)	37/43 (86.1)	16.98 (15.25)	86.4 (29.1; *N *= 41; 75.6%)	12.7 (5.9)	10.5 (6.4)	(*N *= 0)	5.3 (2.9)

All affected subjects	181	155:26 (5.96:1)	106/168 (63.1)	14.64 (12.51)	101.9 (33.5; *N *= 167; 75.5%)	19.5 (7.5)	15.2 (6.1)	12.3 (2.3; *N *= 26)	6.3 (2.7)

All relatives with no ASD diagnosis	448	211:237(0.9:1)		37.30 (20.49)	28.7 (24.0; *N *= 351; 22.5%)				

* 11 cases were missing an Autism Diagnostic Interview (ADI); five cases were missing an Autism Diagnostic Observation Schedule.

^† ^Three cases were missing an ADI; no cases were missing an ADOS.

SD, standard deviation; SRS, Social Responsiveness Scale; Restr/Repet, Restricted Interests/Repetitive Behaviors Domain Score

**Figure 1:**
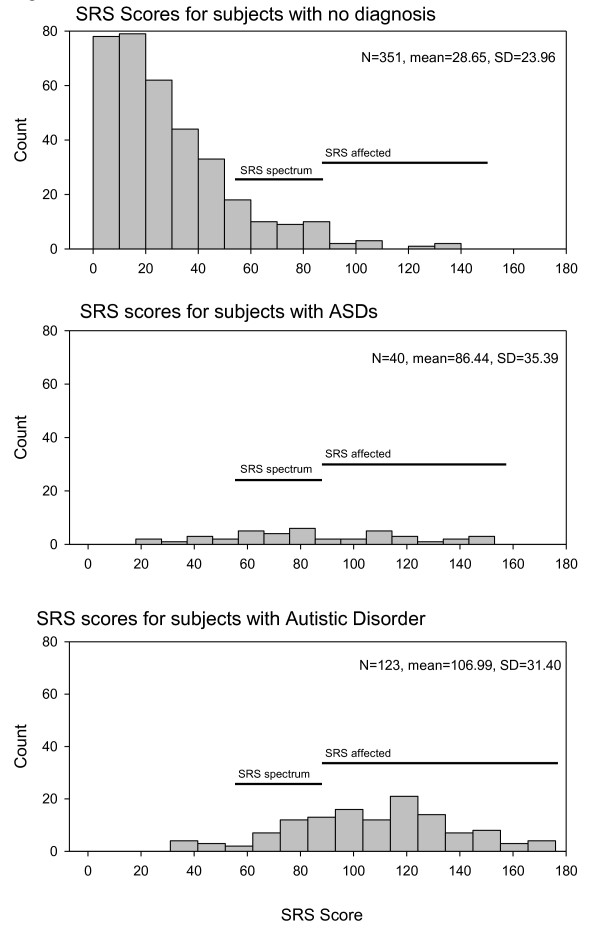
**Histograms of Social Responsiveness Scale scores for subjects in Utah autism pedigrees**.

Regression adjustment of the SRS for age and gender effects resulted in parameter estimates of -0.074 for age and 1.6 for gender (males have higher scores). Figure 1 gives the distribution of SRS scores by diagnostic category. SRS scores were significantly different between autism and ASD (*t *= 3.71, *P *= 0.0003) and between ASD and other relatives (*t *= 10.57, *P *< 0.0001). Among affected subjects, substantial correlations were observed between ADI domain scores and the SRS total score. The highest correlations were observed with the ADI social domain score, with a correlation of 0.60 among ASD subjects and a correlation of 0.46 among subjects with AD. For ASD subjects, 17.1% scored in the normal range on the SRS, 39.0% scored in the spectrum range and 43.9% scored in the affected range. Among subjects with a clinical diagnosis of autism, 5.6% scored in the normal range, 19.1% scored in the spectrum range and 75.4% scored in the affected range. The 95 genotyped spouse pairs showed a significant spouse correlation of 0.33 (*P *= 0.001) using the square root transformed adjusted scores.

**Figure 2:**
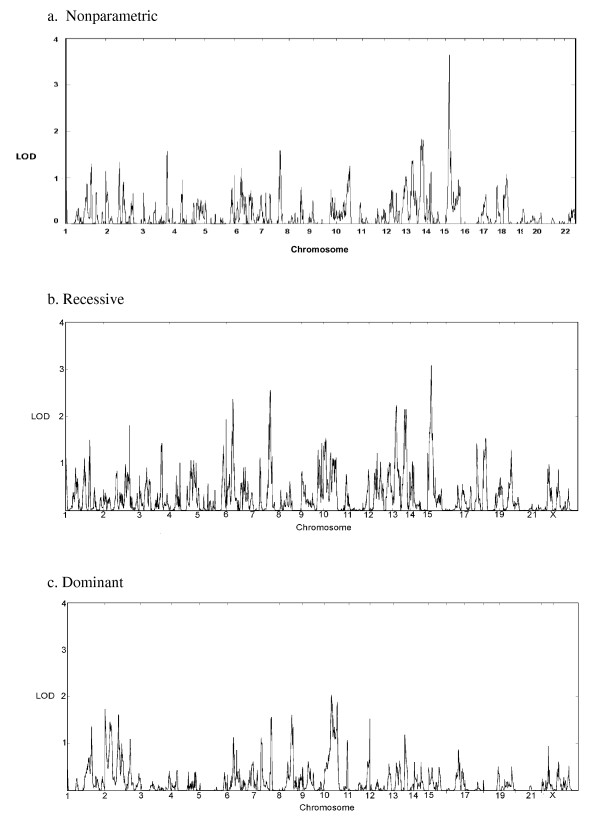
**Genome scan results of Social Responsiveness Scale scores treated as qualitative categories (unaffected, spectrum, and affected) using MCLINK**. (a) The nonparametric analysis does not assume a genetic model; scores for this analysis have been converted to logarithm of odds (LOD) scores for ease of comparison. Analyses were also performed using basic recessive (b) and dominant (c) models with parameters consistent with the prevalence of autism spectrum disorder.

**Figure 3:**
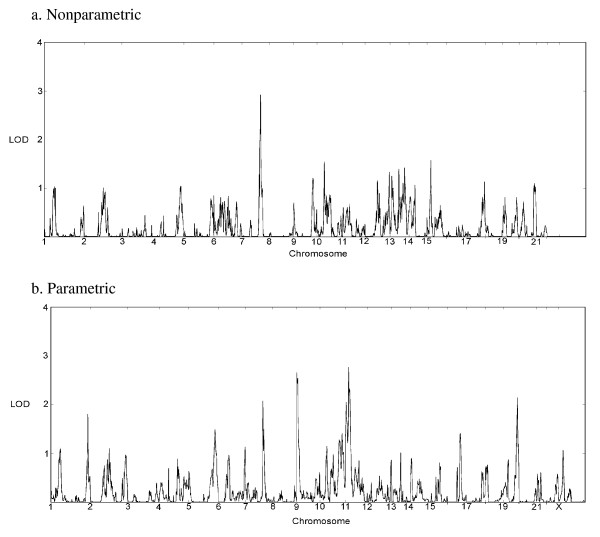
**Genome scan results of quantitative Social Responsiveness Scale (SRS) scores using MCLINK**. The nonparametric analysis (a) does not assume a genetic model, and scores were converted to logarithm of odds (LOD) scores for ease of comparison. The parametric analysis (b), assumes the penetrance vector for an individual is based on their observed trait value and three specified trait distributions for the three genotypes at the disease locus (no risk allele, one risk allele, two risk alleles). These trait distributions are assumed to map onto the SRS score cutoffs (unaffected, spectrum, and affected). The penetrance vector for an individual is the relative probabilities of the trait being observed from the three distributions.

The genome-wide analyses of the data are summarized in Table 3 and in Figures 2 and 3. Note that MCLINK does not provide NPL scores for the X chromosome. Qualitative analyses show substantial overlap with our previous affected-only genome-wide study of these same families [32], in addition to revealing new positive results. We also saw positive findings from our analysis of the quantitative SRS data and replicated the chromosome 11 SRS finding reported by Duvall *et al*. [20].

**Table 3:** Linkage results with *P*-values ≤ 0.0017 (suggestive evidence) for at least one method of analysis from quantitative and qualitative analyses of the total Social Responsiveness Score (SRS).

Chromosome	**Location**^**1**^	**Score (alpha**^**2**^**; *P*-value)**	Phenotype, model
6p25.3	133,969	1.93 (0.40; 0.0014)	Qualitative, recessive

6p22.1	26,261,314	2.36 (0.31; 0.00049)	Qualitative, recessive

*7q31.1-q32.3*	113,934,453	2.07 (0.54; 0.001)	Quantitative, parametric

	129,451,548	2.91 (--; 0.00013)	Quantitative, NPL

	130,291,151	2.55 (0.37; 0.00031)	Qualitative, recessive

9p24.3^3^	593,192	2.66 (0.75; 0.00023)	Quantitative, parametric

10p12.1	27,770,267	2.03 (0.74; 0.0011)	Qualitative, dominant

10q22.1-q22.2	76,287,863	1.88 (0.65; 0.0016)	Qualitative, dominant

11p15.1-p15.4^4^	12,519,296	2.77 (0.44; 0.00018)	Quantitative, parametric

*13q12.3*	30,070,594	2.23 (0.60; 0.00068)	Qualitative, recessive

13q32.1	94,452,168	2.14 (0.85; 0.00085)	Qualitative, recessive

*15q13.3*	31,177,959	**3.64 (--; 0.000021)**	Qualitative, NPL

	31,177,959	3.07 (0.70; 0.000085)	Qualitative, recessive

19q13.43	59,338,102	2.14 (0.71; 0.00085)	Quantitative, parametric

Bold font indicates significant evidence (*P *≤ 0.000048).

*Note: *All nonparametric linkage (NPL) scores have been converted to equivalent logarithm of the odds (LOD) scores. Chromosomal locations indicated in italics overlap our affected-only genome scan using clinical diagnosis [32].

^1 ^Location at the maximum of the peak.

^2 ^Alpha, the estimated proportion of linked families for the MCLINK heterogeneity LOD score, is given for parametric analyses.

^3 ^The peak at 9p24.3 is in the same location as a peak reported in a single family from our sample [35].

^4 ^The peak at 11p15.1-p15.4 replicates previous genome scan results of quantitative SRS scores [20].

**Figure 4:**
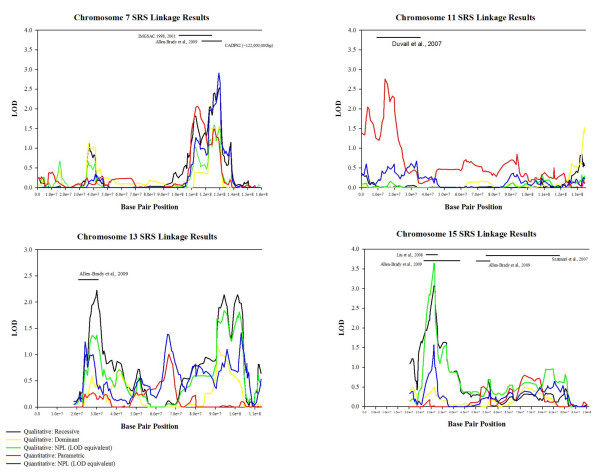
**Detail of levels of detail (LOD) score results on chromosomes 7, 11, 13, and 15**. Scores of nonparametric linkage (NPL) analyses have been converted to LOD scores in order to facilitate comparisons.

Figure 4 shows results for individual chromosomes where peaks overlap previous findings. The most significant peak was found in the MCLINK qualitative nonparametric analysis, with a genome-wide significant LOD score of 3.64 on chromosome 15q13.3 at rs1026752 (31,177,959 bp). Note that we converted all nonparametric linkage (NPL) scores to LOD scores for ease of comparison. The MCLINK recessive model maximized at the same location with a suggestive score of 3.07. This peak extended from about rs1719013 to rs580839 (27,940,000 bp - 32,780,000 bp). This peak was in the same location as the most significant linkage peak from our affected-only genome scan using clinical affection status in these pedigrees [32].

We also saw evidence for linkage in other locations (see Table 3 and Figures 3 and 4). Of these peaks, those on chromosomes 7q31.1-q32.3 and 13q12.3 were also found in our previous genome scan using clinical affection status [32]. The peak at 7q31.1-q32.3 (125,450,000 bp - 130,300,000 bp) showed consistent evidence between quantitative and qualitative analyses. The 13q12.3 peak also showed overlap with our previous clinical diagnosis scan. In the current study, this peak extended from 26,500,000 bp to 33,000,000 bp and was supported by the qualitative recessive analyses. A second apparently distinct peak was found on chromosome 13q32.1 at 94,452,168 bp, also in the qualitative recessive analyses.

Our most significant quantitative peak was on chromosome 7q (LOD = 2.91) in the same region as the evidence from the qualitative analyses. Our next most significant peak was on chromosome 11p15.1-p15.4 spanning a region from 7,300,000 bp to 22,400,000 bp, with a maximum LOD of 2.77 at two adjacent SNPs (rs214101 and rs4757574) from a parametric analysis. The base pair location of our peak (17,246,930 bp - 17,682,950 bp, or 25.59cM - 26.84 cM) is well within one of the most significant peaks previously reported by Duvall *et al*. [20] in their quantitative SRS scan. Other quantitative peaks were found on chromosomes 9p24.3 and 19q13.43. The 9p24.3 peak is in a region previously identified in a clinical diagnosis scan done in our first extended family [44,57].

## Discussion

The present genome-wide linkage scan of SRS scores was performed using 518 relatives from a unique resource of large extended and smaller multiplex ASD families from Utah. We found genome-wide significant linkage on chromosome 15q13.3, and several other suggestive findings. Peaks on chromosomes 7q, 13q and 15q were also found in our previous genome scan of affected-only subjects in these families based on clinical diagnosis [32]. SRS quantitative analyses also replicated a finding on chromosome 11 p reported in a previous genome scan of the SRS [20], providing some evidence for genetic consistency of this measure used as a quantitative trait across studies.

The SRS was developed as a continuous measure of autistic traits, with variation intended to extend across affected subjects and unaffected relatives. Among affected subjects, our data suggest substantial correlations between clinical measures of autism from the ADI and SRS total scores, particularly for the social behavioural domain. ADI and SRS are expected to differ to some degree because ADI scores are based on information from the developmental period between ages 4-5 and also on the most severe presentation through the individual's life, while the SRS focuses on current behaviour. In addition, most clinically affected subjects scored in the spectrum or affected ranges on the SRS, though as expected, we observed a substantial range of scores. Most of the clinically unaffected relatives scored in the normal range on the SRS (87.4%), but considerable variation also occurred in these scores.

In our previous genome-wide scan, all relatives with no clinical diagnosis were considered unknown, so results were based on phenotypic information from 192 genotyped affected cases. Using the SRS in the present study, we added information for 337 clinically unaffected relatives, though 11 clinically affected subjects from the previous scan were missing SRS scores and were not included here. A small percentage of clinically diagnosed relatives (*N *= 14) scored within the normal range on the SRS and therefore also contributed different information to the current scan. Our families also replicated previous evidence for significant SRS spouse resemblance (*r *= 0.33, *P *= 0.001) [17].

The new information provided by the SRS in these families revealed additional findings beyond our previous affected-only clinical diagnosis scan [32]. Not surprisingly, given the overlap in classification of affected subjects based on the SRS or the ADI/ADOS, the best overlap in findings between the current study and our previous study resulted when the SRS was treated as a qualitative trait. The use of a SRS-defined spectrum category and the addition of information from other clinically unaffected relatives provide the primary difference in findings between this study and our previous clinical diagnosis scan. The investigation of the whole range of quantitative information produced additional results, which again is perhaps not surprising. We used parametric quantitative methods in an effort to increase the similarity between qualitative and quantitative analyses. We realize that multiple tests may have increased our chances of a false positive result. If we conservatively assume that models are uncorrelated, our significance thresholds would be adjusted by log_10_(5) = 0.7 LOD score units [58] for these five tests: dominant, recessive, NPL qualitative tests and parametric and NPL quantitative tests.

Our genome-wide significant result on chromosome 15q13.3 was supported by the qualitative NPL and recessive models and some lesser evidence also by the quantitative analysis (see Figure 4). In our previous genome scan our best results also occurred on chromosome 15q in three locations that may represent a single peak (29,459,872 bp, 36,837,208 bp, and 55,629,733 bp) [32]. Liu *et al*. [27] have also reported evidence for linkage in this region (LOD = 4.01 at 15q13.3-q14).

The region on chromosome 7q31.3-32.3 also occurred in our previous clinical diagnosis scan, and was supported consistently across analysis models. This peak (125,450,000 bp - 130,300,000 bp) has been implicated previously by other studies [59-63]. Our findings suggest that SRS information strengthened this peak over the 7q peak in previous clinical diagnosis scan (LOD = 1.97), though results here are still only suggestive (LOD = 2.91). We note that the CNTNAP2 gene of recent interest in autism [64] is at 145,444,386 bp, downstream of our peak. The final peak showing overlap with our clinical diagnosis scan was on 13q12.3. A second downstream peak at 13q32.1 appeared in the current SRS study that was not evident in the clinical diagnosis scan. While some studies have reported positive findings on chromosome 13 for autism, attention has focused on the neurobeachin gene (NBEAL2) [65], which lies in between our two regions and is, therefore, not supported by our data.

Other peaks were observed on chromosomes 6 and 10. The regions on chromosome 6p25.3 and 6p22.1 have appeared in schizophrenia studies [66-68] and also in bipolar disorder [69,70]. The region also harbors a copy number variation (CNV) at 27,827,354-28,119,631 implicated in autism [71]. Interestingly, chromosome 10p12.1 and 10q22.1-q22.2 also has been reported for schizophrenia and bipolar disorder in many of the same samples that showed chromosome 6 findings [69,72-74]. Somewhat strikingly, two genome-wide associations with attention deficit and hyperactivity disorder (ADHD) have also been found on chromosome 10p12.1 at 27,672,044-27,694,523 and 10q22.1 at 72,126,813-72,120,519 [75]; both findings are quite close to our linkage peaks. Additional evidence exists in two other samples of an association between ADHD and autism using the SRS [76,77].

A quantitative analysis also revealed findings of interest. While results using the quantitative range of the SRS often paralleled the qualitative findings, there were interesting exceptions. We note that while we used the same genetic analysis software, the variation in the trait was treated quite differently, so direct overlap of findings would not necessarily be expected. For the quantitative analysis, we also note that adjusting for age and gender may have been a conservative adjustment, as clinical affection status is associated with age and gender. As a result of this adjustment, our quantitative results are unlikely to be due to behaviours that change with age or behaviour specific to gender. Our highest quantitative peak was on chromosome 7 in the same region as described above. Our next highest quantitative peak was on chromosome 11p15.5-p15.4, spanning a region from 7,300,000 bp to 22,400,000 bp (12.11 - 37.63 cM). This peak replicates a previously reported SRS peak from 15-45 cM found in an analysis of quantitative SRS scores [20]. Liu *et al*. [27] also found linkage in this region, primarily using a phenotype of delayed phrase speech, but with supporting evidence also from other phenotypes. A previous genome scan of clinical diagnosis in 1181 multiplex families also identified this region [78]. On chromosome 19q13.4, our quantitative evidence spanned a region from 57,150,000 bp to 63,780,000 bp. This region overlaps with previous autism linkage evidence [79] and also has several CNVs associated with autism [71].

Finally, we observed peaks in two other regions previously identified in our families in scans using affection status. We observed a quantitative peak on chromosome 9p24.3, from the telomere to about 4,330,000 bp. This peak has been previously reported for obsessive compulsive disorder (OCD) [80,81]. The glutamate transporter genet SLC1A1 is near this region (4,480,000 bp - 4,580,000 bp), and has shown some association with OCD [82,83].

While it is important to seek replication of these findings in independent samples, we intend first to pursue these findings within our sample families. It is possible that findings may be unique to our family sample; indeed, a rare genetic finding for autism would be our expectation. However, if we can find genetic variation responsible for these statistical findings, the results may still provide relevant evidence regarding biological mechanisms and genetic pathways implicated in autism. Our family data will allow us to investigate segregation of the genetic variants responsible for these peaks and to determine more detailed associations with phenotypes related to autism. The extended family design allows a more powerful tool to investigate rare genetic variants in relation to phenotype than the genome-wide association study design.

## Conclusions

In summary, our results highlight three important points. First, using a quantitative analysis, we replicated a previously reported SRS peak on chromosome 11 peak found in the AGRE family sample [20]. This replication supports the SRS as a robust measure, as replication occurred across independent samples, and in spite of differences in study design and measurement. Our sample included large extended pedigrees and more than half the SRS scores in our sample were on adults; the Duvall *et al*. study [20] included only measurements on children. Second, our genome scan of SRS scores revealed several peaks that were the same as those found in our previous affected-only genome scan of clinical diagnosis [32]. These peaks in our Utah families are therefore robust to the inclusion of data from clinically unaffected relatives and to the analysis of autism measured in a manner quite different from the traditional structured interview and observation methods. This study supports our previous ASD linkage findings and suggests the SRS may be a reasonable surrogate measure for genetic analysis of ASD, though the SRS does not provide the depth of information used in a clinical setting. Finally, both qualitative and quantitative analyses of SRS scores revealed linkage peaks not observed using clinical affection status in our own previous pedigree studies, but often overlapping with other evidence from autism, ADHD, OCD, schizophrenia and/or bipolar studies. These results indicate that the additional information available from clinically unaffected relatives and the information in the SRS itself may reveal locations for autism susceptibility genes that would not otherwise be detected.

## Abbreviations

AD: autistic disorder; ADHD: attention deficit and hyperactivity disorder; ADI-R: Autism Diagnostic Interview-Revised; ADOS-G: Autism Diagnostic Observation Schedule-Generic; ASD: autism spectrum disorder; BAP: broader autism phenotype; CIDR: Center for Inherited Disease Research; CNV: copy number variation; HLOD: heterogeneity logarithm of the odds (LOD); IRB: Institutional Review Board; LD: linkage disequilibrium; MCMC: Markov chain Monte Carlo; NPL: nonparametric linkage; OCD: obsessive compulsive disorder; SCQ: Social Communication Questionnaire; SNP: single nucleotide polymorphism; SRS: Social Responsiveness Scale; UPDB: Utah population database.

## Competing interests

HC, WMM, and JSM received partial salary support from Lineagen Inc. (www.lineagen.com) from 12/1/07 to 12/31/08.  This salary support is not ongoing.

## Authors' contributions

HC conceived the study, participated in its design, directed the statistical analyses and drafted the manuscript. MEV verified the accuracy of the phenotype data and made contributions to the manuscript. RJR assisted with the interpretation of the results and helped draft the manuscript. NJC, DSC, and KAB helped with the statistical analyses and helped draft the manuscript. JSM confirmed the research diagnoses of ASD, supervised the collection of all phenotype data and made significant contributions to the interpretation of results. WMM participated in the design of the study and helped draft the manuscript. All authors read and approved the final manuscript.
